# Comparative transcriptome analysis of microsclerotia development in *Nomuraea rileyi*

**DOI:** 10.1186/1471-2164-14-411

**Published:** 2013-06-19

**Authors:** Zhangyong Song, Youping Yin, Shasha Jiang, Juanjuan Liu, Huan Chen, Zhongkang Wang

**Affiliations:** 1 Genetic Engineering Research Centre, School of Life Science, Chongqing University, Chongqing 400030, China

**Keywords:** Transcriptome, Oxidative Stress, Microsclerotia, *Nomuraea Rileyi*

## Abstract

**Background:**

*Nomuraea rileyi* is used as an environmental-friendly biopesticide. However, mass production and commercialization of this organism are limited due to its fastidious growth and sporulation requirements. When cultured in amended medium, we found that *N*. *rileyi* could produce microsclerotia bodies, replacing conidiophores as the infectious agent. However, little is known about the genes involved in microsclerotia development. In the present study, the transcriptomes were analyzed using next-generation sequencing technology to find the genes involved in microsclerotia development.

**Results:**

A total of 4.69 Gb of clean nucleotides comprising 32,061 sequences was obtained, and 20,919 sequences were annotated (about 65%). Among the annotated sequences, only 5928 were annotated with 34 gene ontology (GO) functional categories, and 12,778 sequences were mapped to 165 pathways by searching against the Kyoto Encyclopedia of Genes and Genomes pathway (KEGG) database. Furthermore, we assessed the transcriptomic differences between cultures grown in minimal and amended medium. In total, 4808 sequences were found to be differentially expressed; 719 differentially expressed unigenes were assigned to 25 GO classes and 1888 differentially expressed unigenes were assigned to 161 KEGG pathways, including 25 enrichment pathways. Subsequently, we examined the up-regulation or uniquely expressed genes following amended medium treatment, which were also expressed on the enrichment pathway, and found that most of them participated in mediating oxidative stress homeostasis. To elucidate the role of oxidative stress in microsclerotia development, we analyzed the diversification of unigenes using quantitative reverse transcription-PCR (RT-qPCR).

**Conclusion:**

Our findings suggest that oxidative stress occurs during microsclerotia development, along with a broad metabolic activity change. Our data provide the most comprehensive sequence resource available for the study of *N*. *rileyi*. We believe that the transcriptome datasets will serve as an important public information platform to accelerate studies on *N*. *rileyi* microsclerotia.

## Background

*Nomuraea rileyi* is a well-known fungal pathogen thriving in a range of obligate hosts, particularly the noctuids. It affects polyphagous species of *Heliothis*, *Spodoptera*, *Pseudoplusia*, *Trichoplusia*, *Plutella*, and *Rachiplusia*[1,2], and hence, can be used as a mycoinsecticide. However, the lack of reliable cost-effective protocols for mass production of this entomopathogenic fungus limits its commercialization. The present mass productions methods are not cost effective and require special growth conditions (stimulatory light and maltose) for sporulation [3]. As a result, there is a need to find an active agent for commercial production of *N*. *rileyi*. Microsclerotium (MS), with a diameter of 200–600 μm, is a pseudoparenchymatous aggregation of hyphae, comprising only a few cells. It is produced by many phytopathogenic fungi for persistence in the soil and decaying plant material. The structures of fungal phytopathogen, including the infective and spreading structures, help them to survive for long periods in the environment [4,5]. MS production was successfully induced in phytopathogenic fungus [6,7] and entomopathogenic fungus *Metarhizium anisopliae*[8] during submerged liquid culture fermentation. Our laboratory successfully realized MS production by *N*. *rileyi* on liquid amended medium (AM), which exhibited insecticidal efficacy on *Spodoptera litura*, and thus can be used as an active agent (data not shown).

The morphological events that occur during microsclerotia production have been studied extensively [9,10]. A mature MS has been observed to exhibit three distinct layers: a pigmented rind, a thin-walled cortex, and a large central medulla [11,12]. During the initial stages of development, the hyphae swell and aggregate. The aggregations subsequently enlarge and the pigment emerges. In the final phase, the pigment gets deposited within the cell wall. The mature microsclerotia can disperse in the environment as persistent infective propagules, affecting a broad range of herbaceous and woody hosts [13].

The potentially important genes in microsclerotia development have already been investigated. Two expressed sequence tag (EST) libraries developed from different cultures of a tomato isolate strain *Verticillium dahliae*, were found to exhibit the most unique sequences, of which 55% had protein sequences similar to those presented in the database [14]. Based on the EST databases, *VDH1*, a hydrophobin gene, was confirmed to be involved in microsclerotia development [15,16]. Subsequently, using the *Agrobacterium tumefaciens*-mediated transformation (ATMT) method, the role of the gene in microsclerotia development was identified. In addition to targeted gene disruption by homologous recombination, the ATMT has been successfully exploited for large-scale forward genetic screen to create insertional mutants. A gene that encodes glutamic acid-rich protein and affects microsclerotia formation and pathogenicity was identified by the ATMT [17]. It has been reported that the physiological and morphological differences during microsclerotia development should be, at least in part, due to numerous genes expressed at various development stages [18-21]. Transcript profiling is an important strategy for studying the expression of large genes. Although EST sequencing has been used for the detection of reference transcripts, it has some inherent limitations, such as low throughput, high cost, and long experimental cycle. Recently, next-generation sequencing (NGS) technology has emerged for high-throughput sequence determination, which is quite economical [22]. The RNA-Seq technology has enabled us to investigate the transcriptome for various gene expression studies without reference genome sequences [23].

In the present study, the NGS technology was used to examine the transcriptome of MS of *N*. *rileyi*. We constructed two libraries sequenced using Illumina HiSeq™ 2000 based on *N*. *rileyi* cultures grown in liquid AM and minimal medium (MM), which produced abundant MS and mycelium, respectively. Subsequently, we analyzed the expression of 32,061 unigenes, found the differentially expressed genes related to MS development, compared the up-regulation of the expression of unigenes in the AM libraries, and validated some unigenes based on different development stages by using RT-qPCR. The two assembled and annotated transcriptome sequences provide an invaluable resource for the identification of *N*. *rileyi* involved in MS development.

## Results

### Illumina sequencing and reads assembly

To comprehensively analyze the induced MS gene expression profile at different external conditions, two cDNA samples were prepared from the mycelia sphere, cultured on MM, and from the developing MS, cultured on AM, and then sequenced using the Illumina sequencing platform. A total of 26 million clean sequencing reads with an average length of 90 bp were generated, respectively. An overview of the sequencing and assembly is outlined in Table 1.The mean contigs size of the sample from MM treatment was 648 bp, with lengths ranging from 101 to 4750 bp, while that of the sample from AM treatment was 536 bp, with lengths ranging from 101 to 4234 bp (Table 1). The size distribution of the contigs is shown in Additional file 1: Figure S1A.

**Table 1:** Summary of the sequence assembly after Illumina sequencing

	**MM**	**AM**
Total reads	26,039,456	26,161,338
Total nucleotides	2,343,551,040	2,354,520,420
Average read length	90 bp	90 bp
Total contigs	48,744	49,221
Mean length of contigs	648	536
Total unigenes	34,231	32,520
Mean length of unigenes	860	738
Total all-unigenes	32,061	
Mean length of sequences	933	
GC percentage	53.80%	54.21%
N percentage	0.00%	0.00%
Q20 percentage	94.11%	93.80%

A total of 32,061 sequences were assembled, with an average of 933 bp and an N50 of 1522 bp (Table 1). The size distribution of those sequences is shown in Additional file 1: Figure S1. Among the sequences, 19,788 were longer than 500 bp, 11,626 were longer than 1000 bp, and 3896 were longer than 2000 bp (Additional file 1: Figure S1B).

### Functional annotation

All-unigenes annotation provides functional information, including protein sequence similarities, clusters of orthologous groups (COG), gene ontology (GO), and Kyoto Encyclopedia of Genes and Genomes pathway database (KEGG) pathway information. For annotation, the sequences were compared with the protein databases (NCBI Nr, SwissProt, KEGG, and COG) using BLASTX (E-value <10^−5^), and the protein functions were predicted from the annotations of the most similar proteins. The sequences were first searched using BLASTX against the NCBI Nr database, and 20,919 sequences (65.25%) had hits that exceeded the E-value cutoff (Additional file 2: Figure S2). A total of 13,385 sequences (41.75%) were identified from the SwissProt database.

**Figure 1:**
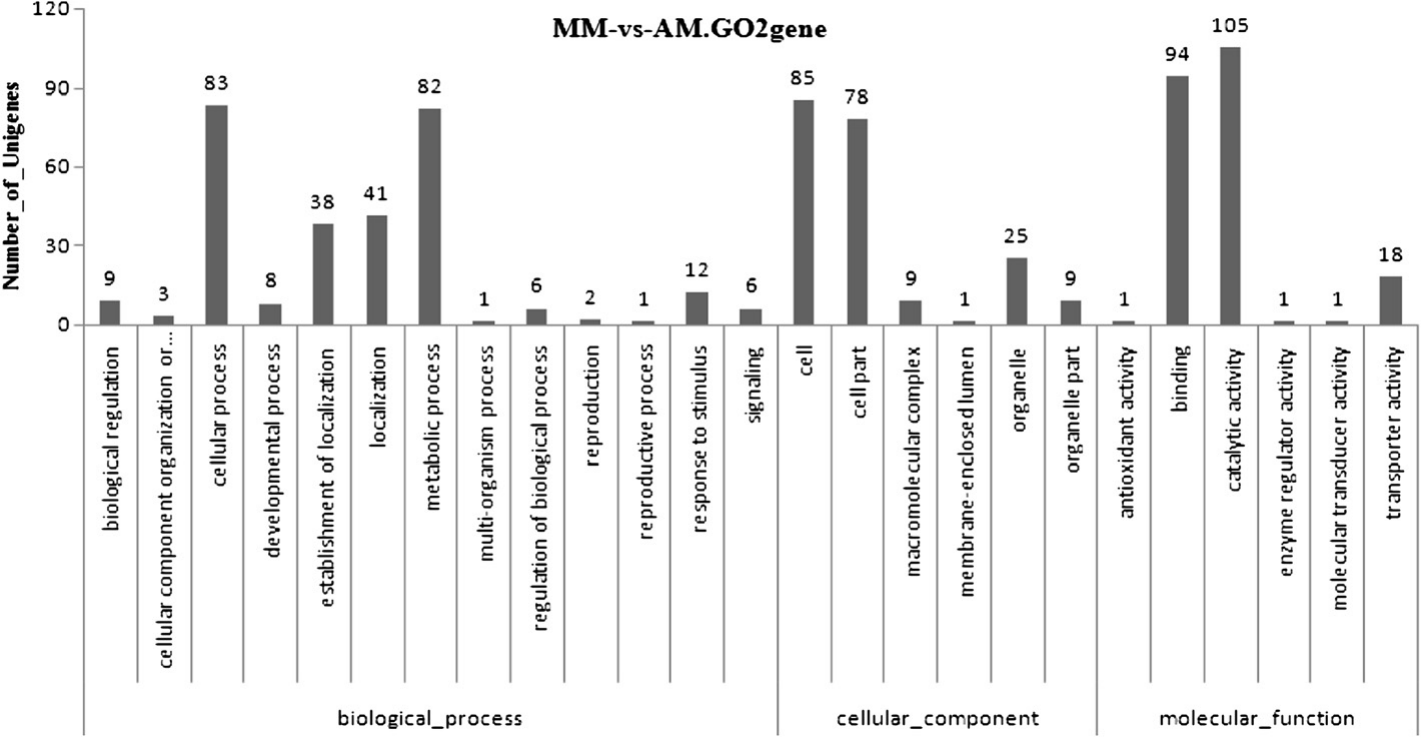
**Histogram presentation of Different Expression Gene Ontology classification.** The results are summarized in three main categories: biological process, cellular component, and molecular function. The *y*-axis indicates the number of genes in a category.

GO analysis was conducted using Blast2go software (http://www.blast2go.com/b2ghome) [24]. A total of 5928 sequences was assigned at least one GO term for describing the biological processes, molecular functions, and cellular components. InterProScan output file was input into the WEGO software (http://wego.genomics.org.cn) [25], and GO annotations were plotted (Additional file 3: Figure S3). To further predict the genes with different expression levels (AM vs. MM), the GO functional analysis was carried out on the differentially expressed genes [26]. The differentially expressed genes were defined as those with false discovery rate (FDR) ≤ 0.001 and ratio of reads per kilobase per million (RPKMs) > 2. Among the differentially expressed genes, a total of 719 sequences was categorized into 25 functional groups (Figure 1).Of these, the dominant terms were “catalytic activity”, “binding”, “cell”, “cellular process” and “metabolic process”, which indicated that these genes were enriched in the two transcriptome libraries.

**Figure 2:**
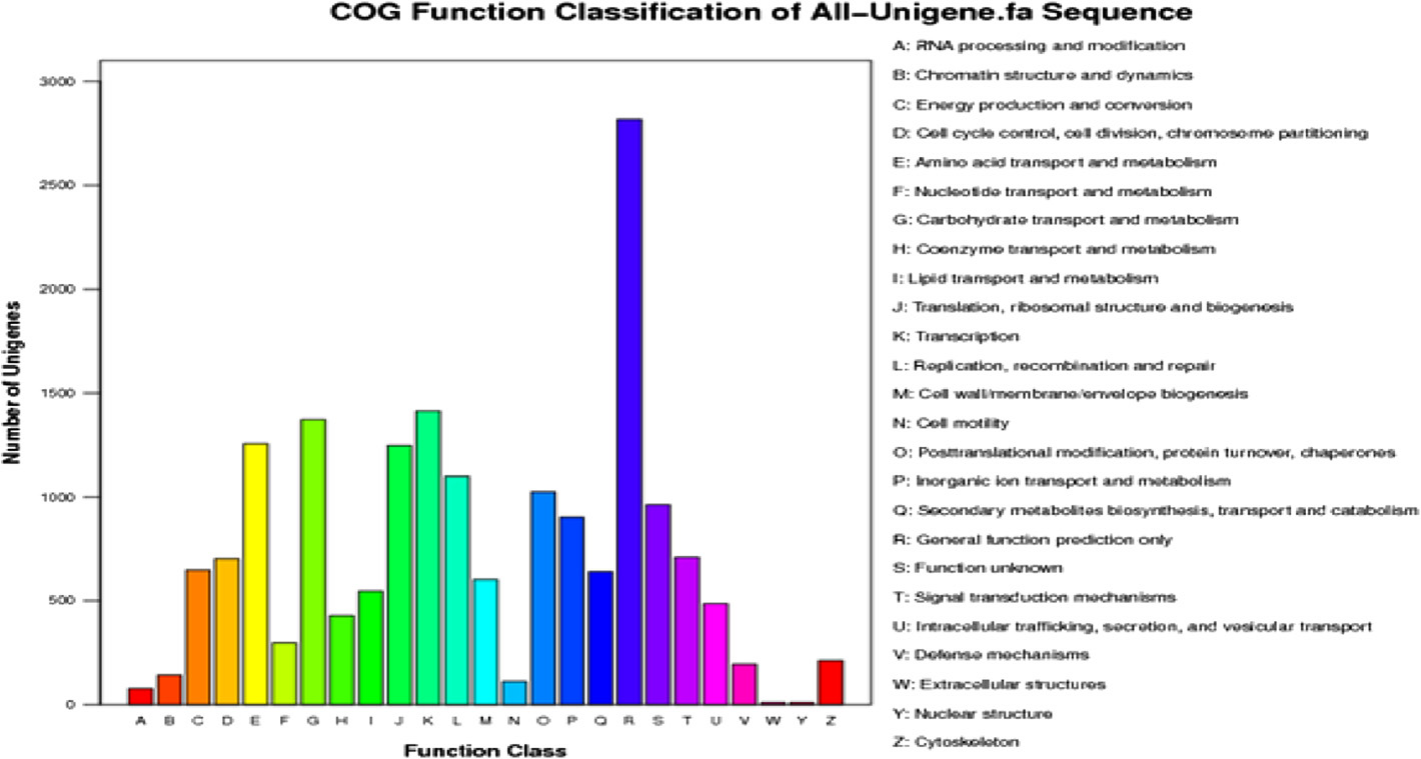
**Histogram presentation of COG classification.** Out of 20,919 nr hits, 17,891 sequences have COG classification among the 25 categories. The *y*-axis indicates the number of sequence in a category.

The unigenes were aligned to COG database to predict and classify possible functions. A total of 17,891 sequences was distributed to 25 COG categories (Figure 2). Among the 25 COG categories, “general function prediction only” represented the largest group (2818; 13.47%), followed by “Transcription” (1412; 6.75%), “Carbohydrate transport and metabolism” (1371; 6.55%), “Amino acid transport and metabolism” (1255; 5.99%), and “Translation, ribosomal structure and biogenesis” (1246; 5.96%). The smallest group was “Extracellular structures” (9; 0.04%) and “Nuclear structure” (9; 0.04%).

The 20,919 nr hits were mapped to the reference canonical pathways in KEGG. In total, 12,778 sequences were assigned to 165 KEGG pathways. To identify the biological pathways that are active in different ways among the two treatments, a total of 1888 differentially expressed sequences was assigned to 161 KEGG pathways. With significant enrichment pathways, we could discern the main biochemical and signal transduction pathways. After multiple testing corrections, we chose pathways with Qvalue  ≤ 0.05 as significantly enriched among the differentially expressed genes. In total, 26 significant enrichment pathways were detected. The enrichment pathways that were most represented were the biosynthesis of secondary metabolites (305 members), metabolic pathways (721 members), and microbial metabolism in diverse environments (300 members). These annotations are valuable resource for the comparison of processes, functions, and pathways in MS research.

### Detection of upregulated genes related to MS development

Following AM and MM treatments, a total of 4808 differentially expressed unigenes were identified. Among the 4808 unigenes, 1538 were up-regulated and 322 were induced to express during AM treatment. Sequence analyses revealed that out of the 1538 unigenes, 691 (44.93%) showed no homology with known sequences and were classified as “no hits”. Among the 322 unigenes, 165 (51.24%) were found to have known functions. We identified a number of unigenes that were induced during AM treatment as well as expressed in the enrichment pathways, such as bifunctional P-450:NADPH-P450 reductase (unigene24005), FAD binding domain protein (unigene23494), glyceraldehyde 3-phosphate dehydrogenase (unigene23268), heat-shock protein (unigene23729, unigene23337, unigene22930, and unigene22875), SUR2-type hydroxylase/desaturase (unigene23801), delta-9 desaturase isoenzyme B (unigene23839), acyl-CoA desaturase (unigene23046), and NADP-dependent mannitol dehydrogenase (unigene23414). Although these unigenes were noted to play different roles in various metabolisms, they were found to be commonly involved in oxidative stress detoxification. This indicates that oxidative stress may occur during MS development [27,28].

### RT-qPCR validation

To confirm the results of the Solexa/Illumina sequencing, the following 17 unigenes, up- regulated or uniquely expressed following AM treatment, were selected for RT- qPCR assays: superoxide dismutase (SOD; unigene5568), catalase (CAT; unigene2410), heat-shock protein (SSC1; unigene23729), glutathione synthase (GS; unigene5050), glutathione S transferase (GSTs; unigene13979), glutathione reductase (GR; unigene22615), polyketone synthase (Pks; unigene22495), delta-9 desaturase isoenzyme B (DB; unigene23839), 2OG-Fe (II) oxygenase family oxidoreductase (FO; unigene1892), pyruvate carboxylase (PYC; unigene3898), pyruvate decarboxylase (PDC; unigene9437), aspartate aminotransferase (AST; unigene12484), alanine aminotransferase (MAC; unigene9129), aspartate-tRNA ligase (ARS; unigene18089), prolyl-tRNA synthase (PRS; unigene16762), acetoacetyl-CoA synthase (ACS; unigene22462), and vacuolar ATP synthase catalytic subunit A (ATP-synt A; unigene18015).

**Figure 3:**
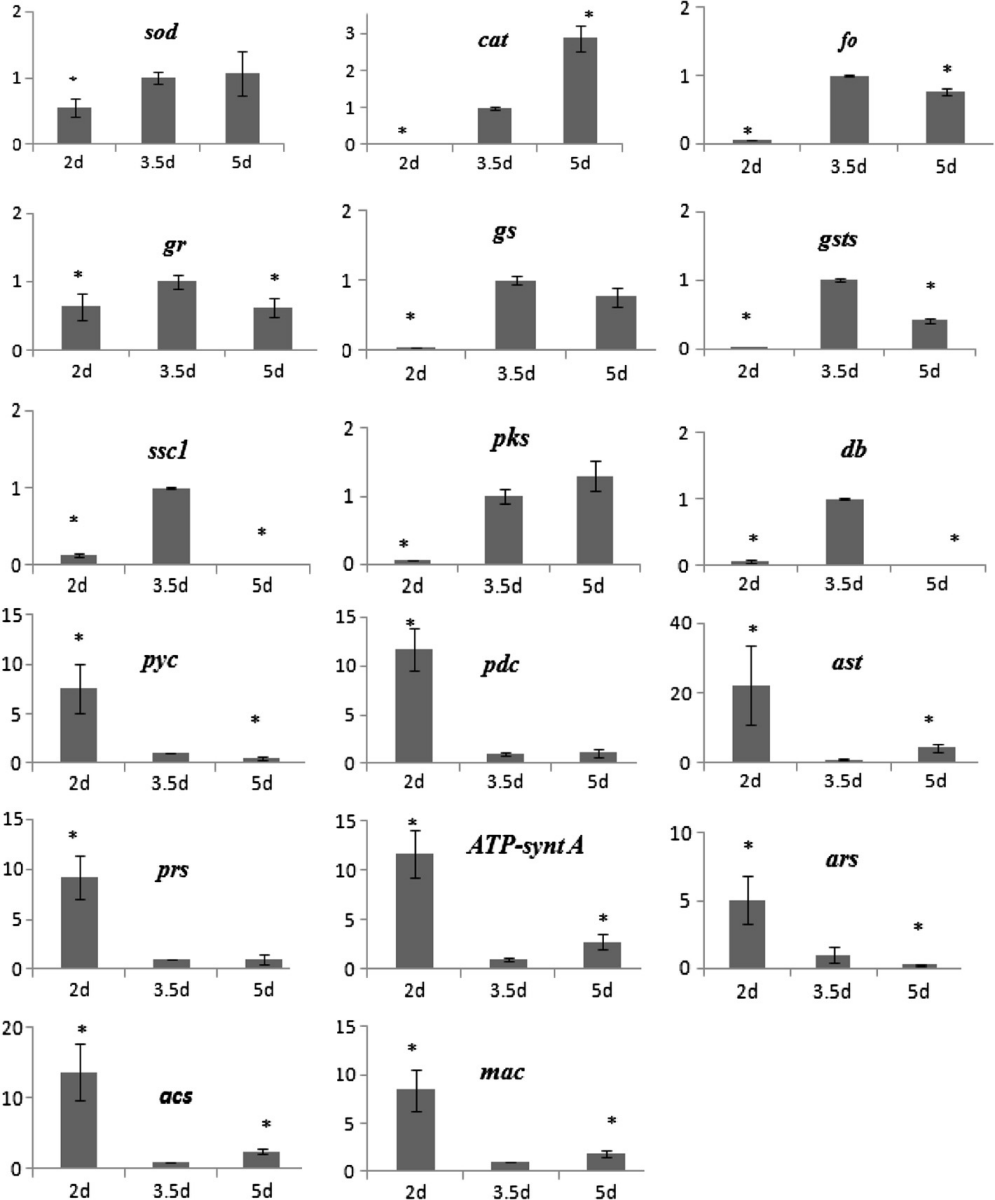
**RT**-**qPCR analyses of 17unigenes during MS development.** Line diagrams represent the expression pattern of selected genes from the two transcriptomes. The expression was analyzed at 2, 3.5, and 5 days. Error bars represent ± SE. Letter over the bars represent statistically significant differences between the expression changes of the genes (statistics were generated based on 3.5day using student *t*-test with *p* < 0.05).

The selected unigenes showed differential expression patterns related to development periods. We found that the RT-qPCR validation of two unigenes (unigene9437 and unigene12484) was not consistent with the sequencing results (Additional file 4: Table S1). Subsequently, the expression of 17genes during MS development was analyzed (Figure 3). Seven genes annotated as related to reactive oxygen species (ROS) detoxification were validated as being highly expressed during MS development (*sod*, *cat*, *fo*, *gr*, *gs*, *gsts*, and *ssc1*). Furthermore, the genes involved in central metabolism were also validated (*pyc*, *pdc*, *ast*, *mac*, *ars*, *prs*, *acs*, and *ATP*-*synt A*), but their expression during MS formation was found to be lower. In addition, the differential expression patterns of genes related to pigment synthesis observed using RT-qPCR was in agreement with those noted by Solexa analyses (*db*, *pks*, and *acs*).

## Discussion

High-throughput mRNA sequencing technology is particularly suitable for gene expression profiling in non-model organisms without prior genome annotation [23]. In the present study, we used NGS technology, based on the Illumina HiSeq™ 2000 platform, obtained 9.2 Gb of coverage with 52,200,794 clean sequencing reads, and identified 32,061 all-unigenes from two libraries with a mean length of 933. Among them, 20,919 (65.25%) unigenes were successfully annotated. Among the two transcriptomes, approximately 4800 unigenes were found to be differentially expressed. The results of the present study expand on those reported in microsclerotia studies based on EST sequencing, and provide more comprehensive genetic and genomic information [14,29].

During the undifferentiated (UD) stage of MS, similar to other phytopathogenic fungi, there is abundant hyphal growth, accompanied by material and energy metabolism. In the present study, numerous related genes were found to be up-regulated during this stage (Figure 3). Furthermore, the natural byproducts of mitochondrial respiration and oxygen consumption were noted to be converted to ROS, such as superoxide anion radical (O_2_^−^). This is the initial step in the formation of ROS by donating a single electron to the oxygen molecule (O_2_), as well as a product of important enzymes such as xanthine oxidase (XO) and NADPH oxidase [30,31]. However, O_2_^−^ radical is not highly oxidizing, and subsequently produces H_2_O_2_ to exert its toxic role. H_2_O_2_ can capture an electron from another O_2_^−^ or free ferrous (Fe^2+^) and cuprous ions, forming hydroxyl radical (OH), which is highly reactive and causes lipid peroxidation; one of the major end products of lipid peroxidation had been shown to be involved in signal transduction [32]. Furthermore, H_2_O_2_ is stable and can cross cell membrane. Thus, although H_2_O_2_ is a harmful metabolic byproduct, it also functions as a ubiquitous intracellular messenger. Different levels of H_2_O_2_ can induce distinct responses within a cell, especially for the proper development and proliferation of cells [25,33,34].

Enzymatic defenses against O_2_^−^ include SOD that dismute O_2_^−^ to H_2_O_2_ in fungi [35], and the organisms defend against H_2_O_2_ mainly by CAT to catalytically decompose it to H_2_O and O_2_[36]. In the present study, unigene5568, annotated as *sod*, was highly expressed in the AM library. In particular, the expression level increased between the development and maturation stages (Figure 3). An earlier study also reported a similar finding during MS development in *Sclerotinia sclerotiorum*[37]. In the present study, maximum consumption of O_2_ was observed at the MS initiation (SI) period (data not shown), demonstrating that O_2_^−^ was continuously produced and the alteration occurred between the UD and SI period. The unigenes (*cat*, unigene11956, unigene13044, unigene13045, and unigene19140) were highly expressed in the AM library, and their expression was the highest during the SI period (Figure 3). Among the basal salts in the AM culture, Fe^2+^ was noted to promote MS biogenesis (data not shown), which could catalyze the formation of OH^.^ radicals. In the present study, the expression of unigene1892 (*fo*) was not only high in the AM library, but was also relatively highest during the SI period (Figure 3). Thus, the generation of ROS may play an important role in the induction of MS formation, as observed in other filamentous fungi [38,39]. Furthermore, enzymes involved in the detoxification of free radicals also appear to have important role during MS development.

Generation of ROS has forced many organisms to devise complex antioxidant defense, such as thioredoxin (TRX), glyceraldehyde 3-phosphate dehydrogenase (G3PDH), and reduced glutathione (GSH). The GSH, in particular, has been shown to be the main non-enzymatic antioxidant defense, helping to maintain the reduced environment of cytosol, and can be used as a substrate to detoxify the cell from oxidant xenobiotic molecules using GSTs to form oxidized glutathione (GSSG) [32]. GR is a ubiquitous flavor enzyme of disulfide reductase family, which catalyzes the NADPH-dependent reduction of GSSG to GSH. These enzymes maintain the balance of the redox couple (GSSG/GSH). The expression of unigenes (*gs*, *gr*, and *gsts*) of functional redox systems involved in the balance of thiol and oxydic glutathione was high in the AM library, and highest during the SI period (Figure 3).This shows the increase in the oxidative stress associated reduced thiol when the undifferentiated mycelium enters the differentiated state [40], which can combat the incremental oxidative stress during MS development. Other stress regulators, such as heat-shock proteins, play important role during oxidative stress, and have well-established roles during different stress conditions in the organism and are up-regulated during stress [41,42]. The unigene (*ssc1*) showed prominent multiple peaks in RT-qPCR and illustrated regulatory activity under oxidative stress (Figure 3).

ROS are not only inevitable byproducts of oxygen metabolism, but also play a role in cellular signaling, which mediates or augments the effects of growth factors, cell differentiation, and programmed cell death [43]. Based on the AM library, the dynamic activity of MS was found to be cell differentiation during the SI period (Figure 1), and the doses of H_2_O_2_ were presumed to reach a certain level (Figure 3) or “sub-poison” concentration [33,34]. The levels of ROS accumulated during stress condition and their subcellular source were noted to determine the expression pattern of specific genes and induction of stress responsive pathways [44]. Much of this response appeared to be designed to decrease energy production and biochemical processes. In this regard, the TCA cycle was reported to be a significant source of reducing equivalents. The regulation of the two genes, *py*c and *pdc*, related to pyruvate carboxylation, was low (5 and 13 folds, respectively; Figure 3), and the induction of genes of amino acid and fatty acid metabolism was 10 and 30 folds (*mac*, *ast*, *acs*), respectively. These findings indicate that low-level metabolism may be important to balance the generation of proton. In addition, several other genes (*prs*, *ars*, *ATP*-*synt A*) were also significantly down-regulated during this period (Figure 3).

The pigment granules were found to be deposited on the rind during *Nomuraea rileyi* MS formation, but not during melanin production [6-8]. An earlier study examined the biosynthetic pathway of the pigment in other fungi [45]. In our transcriptome, the unigene22495, identified as *pks*, was observed to be involved in the biosynthesis of the pigment. The abundance of this gene increased significantly between the SI and MS development (SM) period (Figure 3), and the change in genotype was in accordance with the phenotype. It has been demonstrated that pigment production can be uncoupled from other morphological changes associated with MS development [14,46]. Two unigenes (*acs* and *db*) were noted to be involved in the metabolism of lipid acid. The *db* gene increased significantly during the SI period (Figure 3), indicating that the unsaturated lipid acid participates in resisting oxidative stress. Similarly, the *acs* gene was maintained at high levels during the UD period and remained at certain expression level during other periods (Figure 3), implying that the gene not only takes part in lipid metabolism, but is also related to the biosynthetic pathway of the pigment [45].

In the present study, a large number of differentially expressed genes involved in MS development were found. We analyzed the expression pattern of these differentially expressed genes, particularly, their up-regulation and unique unigenes in the AM libraries. These unigenes were found to primarily code for stress-responsive genes or a part of the cell machinery underlying the biogenesis of various bodies; however, numerous genes with unknown functions were also noted [31].These results indicate that oxidative stress takes place during MS differentiation, and the signaling genes should be further identified. Overall, this study suggests that fungi employ various pathways and regulatory networks of genes in response to different culture conditions, and demonstrated at the genetic level that MS differentiation in *N*. *rileyi* is related to high oxidative stress.

## Conclusion

Using the Illumina sequencing, we surveyed the two transcriptomes of *N*. *rileyi* under different culture conditions, assembled 32,061 unigenes, annotated 20,919 of those unigenes, and found 4808 differentially expressed unigenes among the two libraries. Our findings substantially contribute to the existing sequence resources for *N*. *rileyi*, and will certainly accelerate the research on MS development. In addition, the *de novo* transcriptome analysis of non-model organism without prior genome annotation has again been successfully demonstrated in this study. The genes data will provide a portfolio of candidate genes for further research on the gene expression, genomics, and functional genomics of *N*. *rileyi*.

## Methods

### Strain and growth conditions

*N*. *rileyi* strain was obtained from the Genetic Engineering Research Center, Chongqing, China. The strain was grown on Sabouraud maltose agar fortified with 1% yeast extract (SMAY) for 14 days, under continuous light at 25°C. The conidia suspension, suspended in sterile deionized water with 0.5% Tween 80 (Sigma-Aldrich, America) at a concentration of 1 × 10^8^ conidia/mL, was inoculated in a 100-mL liquid medium (in flask) and incubated in a shaker at 25°C and 200 rpm. The MM (consisting of 40 g/L of glucose, 2.5 g/L of peptone, and 5 g/L of yeast extract) was designed to reflect the nutritional conditions of hypha development. The AM (comprising 40 g/L of glucose, 2.5 g/L of peptone, 5 g/L of yeast extract, 4.0 g/L of KH_2_PO_4_, 0.8 g/L of CaCl_2_.2H_2_O, 0.6 g/L of MgSO_4_.7H_2_O, 0.1 g/L of FeSO_4_.7H_2_O, 37 mg/L of CoCl_2_.6H_2_O, 16 mg/L of MnSO_4_.H_2_O, and 14 mg/L of ZnSO_4_.7H_2_O) contained substances that could induce the development of MS. The development stages, including UD, SI, MS development (SD), and SM, of *N*. *rileyi* were confirmed in 2, 3.5, 4, and 5 days, respectively. Two samples, separately cultured in the liquid MM and AM for 3.5 days, were collected using Whitman filter paper. The mycelia tissues were separated from MS using forceps and washed twice with distilled autoclaved water. Subsequently, the two samples were frozen at −80°C until further use.

### RNA isolation and library preparation for transcriptome analysis

Total RNA was isolated using the TRIzol® reagent (Invitrogen, USA). The RNA samples were then digested using DNase I for 30 min at 37°C to remove potential genomic DNA. RNA integrity was confirmed using the 2100 Bioanalyzer. The mRNA was purified from total RNA using oligo (dT) magnetic beads. Subsequently, the mRNA was fragmented into small pieces using divalent cations under elevated temperature. The cleaved RNA fragments were used for first-strand cDNA synthesis by employing random primers. The second-strand cDNA synthesis was carried out using DNA polymerase I and RNase H. These cDNA fragments were subjected to an end repair process and ligation of adapters. Subsequently, these products were enriched by PCR to create the final libraries (AM and MM).

### Analysis of Illumina sequencing results

The sequencing library was sequenced using the HiSeq^TM^2000 platform (Illumina). Over 26 million sequence raw reads were generated for the analysis. The adaptor fragments were removed from the raw reads to yield the clean reads required for the analysis. *De novo* transcriptome assembly of these short reads was carried out with short reads assembling program –Trinity [47]. Trinity first combined the reads with certain length of overlap to form longer fragments without N, using “N” to represent unknown sequences. These contigs were utilized for further process of cluster using sequence clustering software to form longer sequences without N [48]. These sequences were defined as unigenes. Unigenes from each sample’s assembly were subjected to further sequence splicing and redundancy removal using sequence clustering software to acquire non-redundant unigenes as long as possible. These were designated as all-unigenes.

For further analysis, we used BLASTX (E-value < 10^−5^) to compare between unigenes and protein databases such as NCBI nr, Swiss-Prot, KEGG, and COG, and the best aligning results were used to decide the sequence direction of the unigenes. Unigene annotation provided information regarding the expression and functional annotation of the identified genes. With nr annotation, we used Blast2GO program to obtain GO annotation of the unigenes. After acquiring the GO annotation for every unigene, we used WEGO software to carry out GO functional classification for all-unigenes and understand the distribution of gene functions of the species at the macro level. The COG and KEGG pathways annotation was carried out using Blastall software against COG (http://www.ncbi.nlm.nih.gov/COG) and KEGG (http://www.genome.jp/kegg/) database [49].

### Gene validation and expression analysis

One differentially expressed gene with potential roles in MS development was chosen for validation using RT-qPCR (the primers designed for RT-qPCR analyses are shown in Additional file 5: Figure S2). The samples, cultured in the liquid AM for 2, 3.5, and 5days, were respectively collected, and the mycelia tissues were separated from MS using forceps. Subsequently, the mycelia were washed with sterile distilled water for at least two times. The total RNA was extracted using the TRIzol® reagent (Invitrogen, USA) and purified with RNA purification kit (Qiagen, USA). The first-strand cDNA fragment was synthesized from purified RNA. A total of 17 genes were chosen for RT-qPCR. All the genes were first subjected to RT-PCR analysis, and the gel was run to obtain suitable conditions for RT-qPCR analysis. The RT-qPCR reaction was performed according to the protocol of Green Mastermix (Qiagen, USA). Three biological duplicates of each sample and triplicates of each reaction were acquired. The genes *tef* and *tub* were used for calibration in all the experiments. The expression ratios were calculated from cycle threshold values using the 2^−△△CT^ method.

## Competing interests

The authors declare that they have no competing interests.

## Authors’ contributions

ZKW and YPY designed the study. ZYS and YPY carried out data analysis. ZYS, SSJ, JJL and HC collected samples and carried out RT-qPCR analysis. ZYS, ZKW and HC contributed to the writing of the manuscript. All the authors read and approved the final manuscript.

## Supplementary Material

Additional file 1: Figure S1 Overview of the two *N*. *rileyi* transcriptomes sequencing and assembly. (A) Size distribution of Illumina sequencing contigs. (B) Size distribution of Illumina sequencing unigenes and all-unigenes. Click here for file

Additional file 2: Figure S2 Top BLAST hits from NCBI nr database. The size distribution of the CDS produced by searching all-unigenes sequences against the NCBI nr database using BLASTX (E-value < 10^−5^). Click here for file

Additional file 3: Figure S3 Overview of GO function classification of all-unigenes. Click here for file

Additional file 4: Table S1 The relative expression of the genes between AM and MM transcriptomes. (A) The sequencing results of transcriptome. (B) The results of RT-qPCR between AM and MM treatments. Click here for file

Additional file 5: Table S2 The primers designed for RT-qPCR analysis. Click here for file
