# Supplementary material for: Comparative transcriptome analysis of microsclerotia development in Nomuraea rileyi
Source: BMC Genomics. 2013 Jun 19;14:411. doi: 10.1186/1471-2164-14-411 (PMC3698084; doi:10.1186/1471-2164-14-411)

File S1 **Overview of the two *N.rileyi* transcriptomes sequencing and assembly.**

(A) Size distribution of Illumina sequencing contigs.


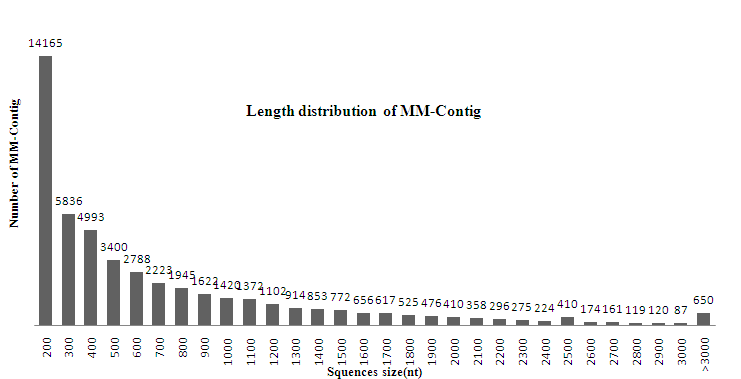


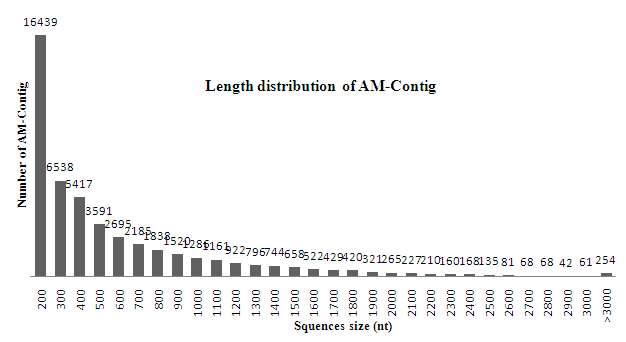


(B) Size distribution of Illumina sequencing unigenes and all-unigenes.


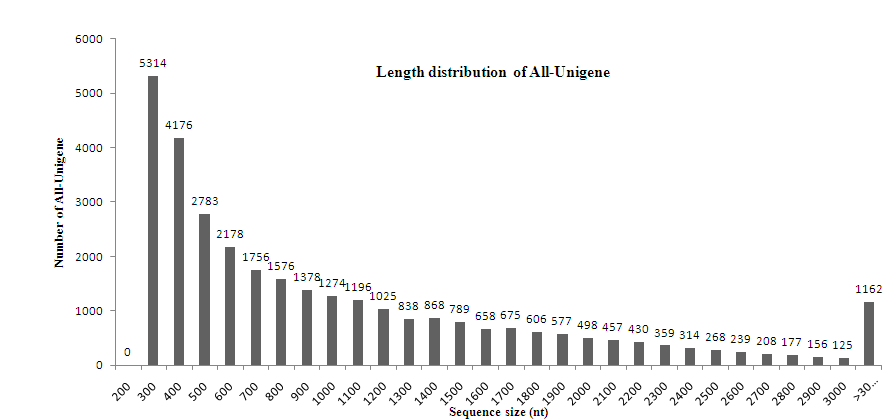


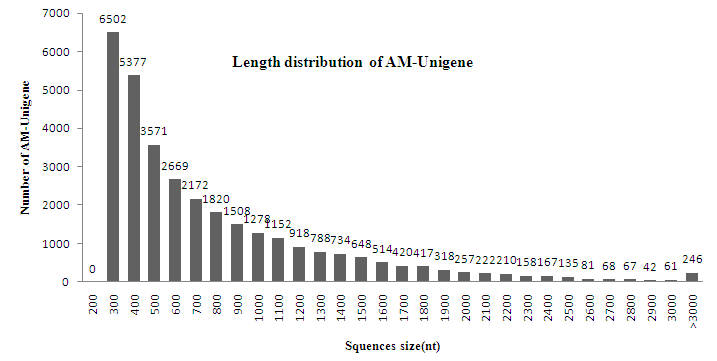


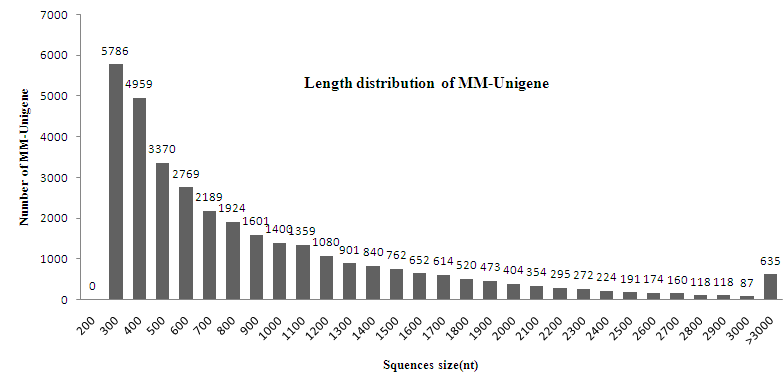

Supplement: Additional file 1: Figure S1 — Overview of the two N. rileyi transcriptomes sequencing and assembly. (A) Size distribution of Illumina sequencing contigs. (B) Size distribution of Illumina sequencing unigenes and all-unigenes. [file 1471-2164-14-411-S1.docx]
