# Supplementary figures and images for: Comparative transcriptome analysis of microsclerotia development in Nomuraea rileyi
Source: BMC Genomics. 2013 Jun 19;14:411. doi: 10.1186/1471-2164-14-411 (PMC3698084; doi:10.1186/1471-2164-14-411)

**File S2 Top BLAST hits from NCBI nr database.**


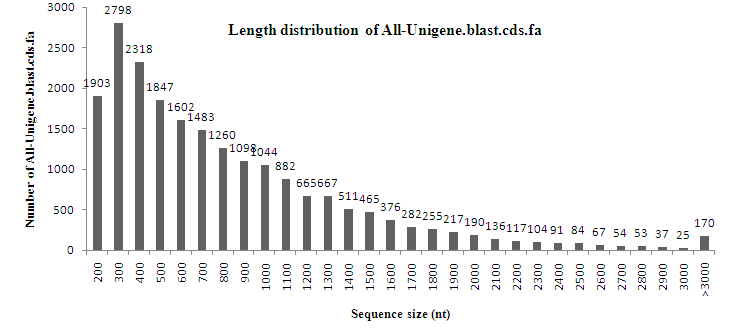

Supplement: Additional file 2: Figure S2 — Top BLAST hits from NCBI nr database. The size distribution of the CDS produced by searching all-unigenes sequences against the NCBI nr database using BLASTX (E-value < 10−5). [file 1471-2164-14-411-S2.docx]

**File S3 Overview GO function classification of all-unigenes.**


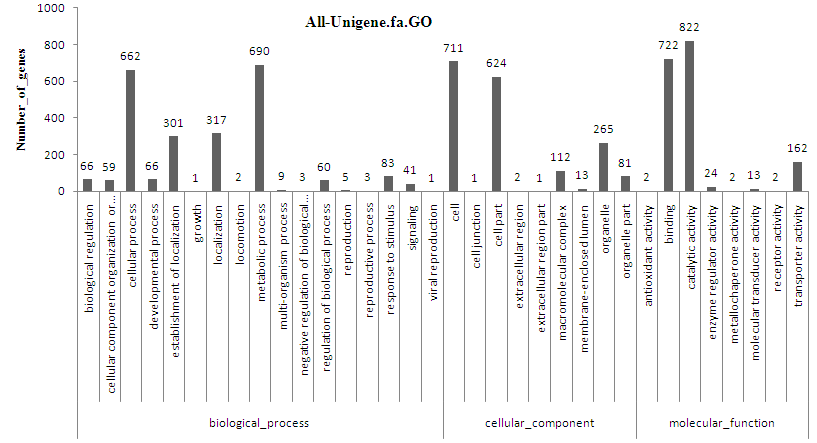

Supplement: Additional file 3: Figure S3 — Overview of GO function classification of all-unigenes. [file 1471-2164-14-411-S3.docx]
