# Supplementary material for: Comparative transcriptome analysis of microsclerotia development in Nomuraea rileyi
Source: BMC Genomics. 2013 Jun 19;14:411. doi: 10.1186/1471-2164-14-411 (PMC3698084; doi:10.1186/1471-2164-14-411)
Supplement: Additional file 4: Table S1 — The relative expression of the genes between AM and MM transcriptomes. (A) The sequencing results of transcriptome. (B) The results of RT-qPCR between AM and MM treatments. [file 1471-2164-14-411-S4.docx]

**File S4 The relative expression of genes between AM and MM transcriptome.**

1. The sequencing results of transcriptome

| geneID | MM RPKM | | AM_RPKM | log2(AIM_RPKM/SDY14_RPKM) | up/down | pvalue | FDR |
| --- | --- | --- | --- | --- | --- | --- | --- |
| *sod* | 18.0168 | | 45.2826 | 1.3296 | Up | 4.17E-76 | 1.30E-74 |
| *cat* | 206.2562 | | 475.4077 | 1.2047 | Up | 0 | 0 |
| *ssc1* | 0 | | 2.2579 | 11.1408 | Up | 2.87E-04 | 8.66E-04 |
| *gs* | 2.7296 | | 7.0654 | 1.3721 | Up | 7.09E-10 | 4.08E-09 |
| *gsts* | 2.3559 | | 5.2284 | 1.1501 | Up | 1.00E-08 | 5.23E-08 |
| *gr* | 0 | | 21.2059 | 14.3722 | Up | 1.45E-38 | 2.56E-37 |
| *pks* | 1.2003 | | 32.4464 | 4.7566 | Up | 8.13E-48 | 1.67E-46 |
| *db* | 0 | | 2.808 | 11.4553 | Up | 6.26E-07 | 2.75E-06 |
| *fo* | 105.0942 | | 2231.73 | 4.4084 | Up | 0 | 0 |
| *pyc* | 20.7377 | | 46.3333 | 1.1598 | Up | 5.44E-20 | 5.45E-19 |
| *pdc* | 2.4884 | | 5.923 | 1.2511 | Up | 1.44E-15 | 1.18E-14 |
| *ast* | 0.282 | | 1.5279 | 2.4378 | Up | 5.21E-08 | 2.54E-07 |
| *mac* | 12.6102 | | 45.9721 | 1.8662 | Up | ######## | ######## |
| *ars* | 0.282 | | 1.5279 | 2.4378 | Up | 5.21E-08 | 2.54E-07 |
| *prs* | 3.0778 | | 10.0617 | 1.7089 | Up | 3.92E-27 | 5.14E-26 |
| *acs* | 1.8345 | | 5.4119 | 1.5607 | Up | 1.35E-19 | 1.33E-18 |
| *ATP-synt A* | | 0.2796 | 1.785 | 2.6745 | Up | 9.61E-23 | 1.07E-21 |

1. The results of RT-qPCR between AM and MM treatments.

Relative expression was analyzed at 3.5d. Error bars represent ±SE. “*” represent statistically significant differences between AM and MM treatment (statistics were generated based on MM treatment using student t-test with p-value <0.05).

| 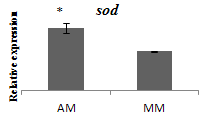 | 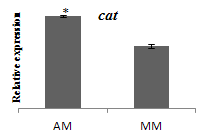 | 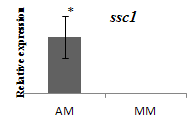 |
| --- | --- | --- |
| 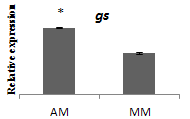 | 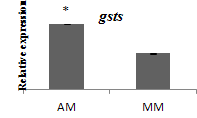 | 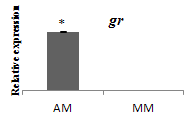 |
| 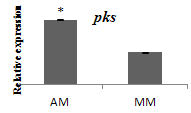 | 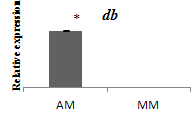 | 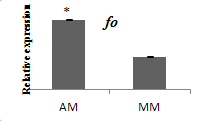 |
| 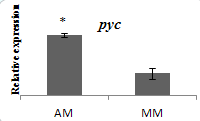 | 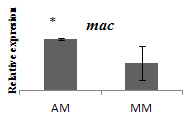 | 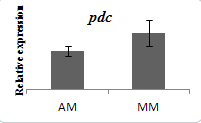 |
| 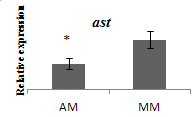 | 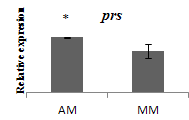 | 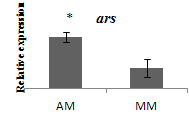 |
| 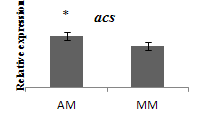 | 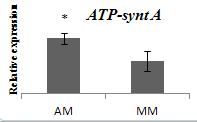 |  |
