# Supplementary material for: Comparative transcriptome analysis of microsclerotia development in Nomuraea rileyi
Source: BMC Genomics. 2013 Jun 19;14:411. doi: 10.1186/1471-2164-14-411 (PMC3698084; doi:10.1186/1471-2164-14-411)
Supplement: Additional file 5: Table S2 — The primers designed for RT-qPCR analysis. [file 1471-2164-14-411-S5.docx]

File S4 the primers designed for RT-qPCR analysis.

| Genes | Name of primer | Sequence |
| --- | --- | --- |
| *sod* | *sod-F* | 5'-AATGCCCAGATCCAAGCTAC-3' |
|  | *sod-R* | 5'-TGATGGTGTTGTGAGGAGGT-3' |
| *cat* | *cat-F* | 5'-ACTGGAAGCTCAACAACCCT-3' |
|  | *cat-R* | 5'-GCGCATCTGGACATTCTTTA-3' |
| *ssc1* | *ssc1-F* | 5’- CCAAGATCATCCTCCACCTT-3’ |
|  | *ssc1-R* | 5’- TGAACTCGGAGTGCTTCTTG-3’ |
| *gs* | *gs-F* | 5'-AAGAAGCAGCTGGACAAGGT-3' |
|  | *gs-R* | 5'-AAACACCGAAAGAGGAGGAG-3' |
| *gr* | *gr-F* | 5’- AACATCACGCACCATGTCTT-3’ |
|  | *gr-R* | 5’- AGGTGTTGCCCTTGTCATCT-3’ |
| *gsts* | *gsts-F* | 5'-GGAGGTACTTGGACCGGATA-3' |
|  | *gsts-R* | 5'-TACGGTTTCCTTTCCGTCTC-3' |
| *pks* | *pks-F* | 5’- AGGCCTATAAAGGCATGCAA-3’ |
|  | *pks-R* | 5’- GCAACTGTCCACCCACATAG-3’ |
| *db* | *db-F* | 5’- TTCTGTGTCAACTCCCTTGC-3’ |
|  | *db-R* | 5’- CGTGGTGGAAGTTGTGGTAG-3’ |
| *acs* | *acs-F* | 5'-CCAGTAGAGGATCTGGCTACG-3' |
|  | *acs-R* | 5'-CAGAAGGGCATTGTCCACTA-3' |
| *fo* | *fo-F* | 5’- TTCCAGACCGTGCTATTCAG-3’ |
|  | *fo-R* | 5’- CGACTTGTCGATGCTGACTT-3’ |
| *pyc* | *pyc-F* | 5’- GAGTACGCCAGCCATATCCT-3’ |
|  | *pyc-R* | 5’-TCTCGGGTGATATGCTGAAC-3’ |
| *mac* | *mac-F* | 5’-CCGCATATCCCTTCTCTGAT-3’ |
|  | *mac-R* | 5’-TCCAACGTGGGAATGATAAA-3’ |
| *pdc* | *pdc-F* | 5’-TGTCAAAGAGAGCGATTTGG-3’ |
|  | *Pdc-R* | 5’-CACGTCTCGATACAATTCCG-3’ |
| *ast* | *ast-F* | 5’-AGATGGTCTGCATCGAAGTG-3’ |
|  | *ast-R* | 5’-CCCGAACTGAATCACGAATA-3’ |
| *prs* | *prs-F* | 5’-GTTGGGAAGCTAAGCAGACC-3’ |
|  | *prs-R* | 5’-TCCAGAATTTCCAGGACCTC-3’ |
| *ATP-synt A* | *ATP-synt A-F* | 5’- CGAGTTTGATGGCAAGAAGA-3’ |
|  | *ATP-synt A-R* | 5’- TTGTCCTACGAGGAAAGGCT-3’ |
| *ars* | *ars-F* | 5’- GGCCATCTTCAAGCTTCATT-3’ |
|  | *ars-R* | 5’- AATGGAAACCATGTGAGCAA-3’ |
| *tef* | *tef-F* | 5’-GTCATCGTCCTCAACCATC-3’ |
|  | *tef-R* | 5’-CAGTCTCAACAGCCTTACC-3’ |
| *tub* | *tub-F* | 5’-GGCAAGGTCGCTATGAAG-3’ |
|  | *tub-R* | 5’-CTGGATGGAGGTAGAGTTAC-3’ |
